# Policy dialogue to improve health outcomes in low income countries: what are the issues and way forward?

**DOI:** 10.1186/s12913-016-1450-2

**Published:** 2016-07-18

**Authors:** Juliet Nabyonga-Orem, Delanyo Dovlo, Aku Kwamie, Ade Nadege, Wang Guangya, Joses Muthuri Kirigia

**Affiliations:** 1 Health Systems and Services Cluster, World Health Organization Regional Office for Africa, B.P. 06 Brazzaville, Congo; 2 University of Ghana, School of Public Health, P.O. Box LG 13, Accra, Ghana; 3 Engage Africa Foundation, 73 Aspen Hills Terr SW, Calgary, AB T3H OP4 Canada; 4 The London School of Economics and Political Science, Houghton St, London, WC2A 2AE UK

**Keywords:** National health policy, Policy dialogue, Health development, Stakeholders

## Abstract

**Background:**

This paper has three objectives: to review the health development landscape in the World Health Organization African Region, to discuss the role of health policy dialogue in improving harmonisation and alignment to national health policies and strategic plans, and to provide an analytical view of the critical factors in realising a good outcome from a health policy dialogue process.

**Discussion:**

Strengthening policy dialogue to support the development and implementation of robust and comprehensive national health policies and plans, as well as to improve aid effectiveness, is seen as a strategic entry point to improving health sector results. However, unbalanced power relations, the lack of contextualised and relevant evidence, the diverse interests of the actors involved, and the lack of conceptual clarity on what policy dialogue entails impact the outcomes of a policy dialogue process. The critical factors for a successful policy dialogue have been identified as adequate preparation; secured time and resources to facilitate an open, inclusive and informed discussion among the stakeholders; and stakeholders’ monitoring and assessment of the dialogue’s activities for continued learning. Peculiarities of low income countries pose a challenge to their policy dialogue processes, including the chaotic-policy making processes, the varied capacity of the actors and donor dependence.

**Conclusion:**

Policy dialogue needs to be appreciated as a complex and iterative process that spans the whole process of policy-making, implementation, review and monitoring, and subsequent policy revisions. The existence of the critical factors for a successful policy dialogue process needs to be ensured whilst paying special attention to the peculiarities of low income countries and potential power relations, and mitigating the possible negative consequences. There is need to be cognisant of the varied capacities and interests of stakeholders and the need for capacity building, and to put in place mechanisms to manage conflict of interest. The likelihood of a favourable outcome from a policy dialogue process will depend on the characteristics of the issue under consideration and whether it is contested or not, and the policy dialogue process needs to be tailored accordingly.

## Background

This paper has three objectives: to review the trend of health development in the World Health Organization (WHO) African Region; to discuss the role of health policy dialogue in improving the harmonisation and alignment to national health policies and strategic plans, and subsequently the health outcomes; and to provide an analytical view of the factors that are critical in realising a good outcome from a health policy dialogue process. Health policy dialogue is increasing in importance as a mechanism for improving governance, yet it remains a phenomenon that is little understood, particularly in low and middle income countries. The current evidence base is thin and fragmented, and this paper debates ways of filling that knowledge gap.

### Health development in the WHO African Region

Health policy dialogue here is understood to be an evidence-informed, deliberative dialogue process among multiple stakeholders for vigorous and comprehensive policy and practice decision-making [1, 2]. The utility of policy dialogue is in its potential to serve as a mechanism for improving governance and building consensus. This is important in a health development context, where multiple actors and global health initiatives support the achievement of health goals.

Despite the registered progress in the attainment of the Millennium Development Goal (MDGs) targets in the countries of WHO African Region, the health indicators still fall below global averages. Life expectancy improved from 50 to 58 years over 1990–2013, but it was lower than the 2013 global average of 71 years [3]. The maternal mortality ratio fell from 960 to 500 deaths per 100,000 live births over 1990–2013 [3], but was notably higher than the 2013 global average of 210 [4]. Under-five mortality came down from 176 to 90 deaths per 1000 births [5] between 1990 and 2013 but was still higher than the global average of 46 [3]. Progress is slow despite the fact that several frameworks to accelerate coverage of health interventions exist. Among these are the Abuja Declaration, which calls for the allocation of 15 % of national budgets to funding for health [6]; the Maputo Plan of Action, which has the goal of ensuring universal access to comprehensive sexual and reproductive health services in Africa [7]; the Paris Declaration and Accra Agenda for Action [8], which define the principles for making development aid more effective; and the Busan Partnership [9], which is committed to improving aid effectiveness through enhanced harmonisation and alignment to country plans, mutual accountability and government leadership of health development.

Efforts to meet the MDGs have led to unprecedented levels of funding, most of this from development partner sources. In fewer than 20 years, approximately 100 global health initiatives (GHIs) have been created to achieve the MDG targets [10]. The result has been increased complexity of governance and financing of health interventions in the WHO African Region. A survey in 37 of the 47 countries in the Region identified 41 GHIs supporting different countries [11]. Among the challenges identified were their lack of alignment with country priorities and financing cycles, lack of financial information for comprehensive planning, weak country leadership, lack of harmonisation across the multiple actors, distrust, and limited use of evidence in decision-making and guidance of the GHI interventions [11]. Relatedly, for countries in the Region, donor funding as a percentage of total health expenditure increased from an average of 2.8 % in 1995 to 11.9 % in 2011 [5], even reaching more than 40 % in some countries in 2013 [5]. Concerns on the need for fiscal alignment and minimising of disruption to the health systems have been raised [12].

The multiplicity of actors and GHIs can provide an opportunity for improving service delivery and eventually health outcomes if the challenges identified are addressed [13]. A well conducted health policy dialogue can provide the opportunity for this. The Sixty-fourth World Health Assembly [14] noted that inclusive policy dialogue with a comprehensive range of stakeholders is critical in increasing the likelihood that national policies, strategies and plans will be appropriately designed and implemented and will yield the expected results.

## Discussion

### Role of health policy dialogue

The role of dialogue for national health policies, plans and strategies is viewed against the backdrop of an increasing focus on evidence-informed decision-making in policy-making and practice. Four key bottlenecks are repeatedly cited as limiting the health sector’s capacity to deliver effective results in terms of improved population health status and outcomes. These relate to the fact that national plans and policies (1) are not sufficiently strategic in terms of long-term health planning; (2) do not sufficiently take into account local and contextualised evidence on priority health issues, along with research evidence on what does or does not work; (3) are not well prioritised in addressing the major causes of ill health; and (4) are decoupled from appropriate and available funds for their implementation [15]. A well-conducted health policy dialogue can facilitate consensus building through promoting stakeholders’ appreciation of one another’s perspectives and increasing participation of stakeholders, including marginalised voices, in the policy process [1]. Health policy dialogue can also facilitate priority setting through developing an understanding of the impact that policies and programmes can have on various groups, as well as serving as an avenue for getting evidence into policy and practice [16]. In addition, policy dialogue facilitates ownership of policies – since they are more responsive to the needs of the stakeholders – and coherence in implementation and monitoring of health plans [1]. Strengthening policy dialogue to support the development and implementation of robust and comprehensive national health policies, plans and strategies, as well as to improve aid effectiveness in line with the principles of the International Health Partnership, is, therefore, an important strategic entry point for improving health sector results.

### Critical factors for a successful health policy dialogue

#### Conceptual clarity

While policy dialogue terminology is gaining currency, a comprehensive definition of the term is still lacking. Scholars have defined policy dialogue in a number of ways, such as “an event where dialogue takes place on a policy question” [17]; a deliberative dialogue and a “group process emphasising transformative and structured discussion” [18]; a recurrent and “integrated part of policy and decision-making processes”; and a process that involves “discussions among stakeholders to raise issues, share perspectives, find common ground, and reach agreement or consensus, if possible, on policy solutions” [1]. Others consider policy dialogue as an “interaction between government and non-governmental organizations at various stages of the policy development to encourage the exchange of knowledge and experience in order to have the best possible public policies” [19]; a “deliberative process (i.e. a structured discussion) which is focused on a policy brief” [20]; a process that “involves people from different interest groups sitting together to focus on an issue in which they have a mutual, but not necessarily common, interest” [21]; and an “open and inclusive dialogue on development policies” [22]. In a study on the role of civil society in policy dialogue, donors defined policy dialogue as “a formal dialogue at government level” while country level stakeholders defined it as a “dialogue between government and civil society and within civil society organisations” [23].

The lack of conceptual clarity may partly explain why not much is understood about policy dialogue. The absence of clarity in the definition of the term in itself prevents the comprehensive appreciation of what policy dialogue entails. Policy dialogue needs to be appreciated as an iterative process that spans the policy action spectrum, from policy formulation to implementation, and covering policy implementation review and monitoring and subsequent policy revisions. In this article we adopt the definition by Dheepa et al. [2] that regards policy dialogue as “a dialogue that is part and parcel of the policy and decision-making processes, intended to contribute to developing or implementing a policy change following a round of evidence-based discussions/workshops/consultations on a particular subject”. This definition captures factors that are particularly important in low income countries, where donors play a significant role in health development. Policy dialogue based on evidence can lead to consensual policies that are supported by stakeholders, especially on issues that may be polarising, considering that stakeholder interests vary. Whilst we acknowledge that policy dialogue is not an entirely new concept given that policy consultations among stakeholders have been going on in line with the Paris Declaration, in our treatment of health policy dialogue we emphasise some aspects, among which are the iterative and two-way nature of the process, implying that although it is led by the government, it involves discussions among stakeholders; the fact that it is not a one-off event but spans the whole spectrum of policy development, implementation and monitoring; and the fact that it allocates evidence a central role in the dialogue.

**Fig. 1:**
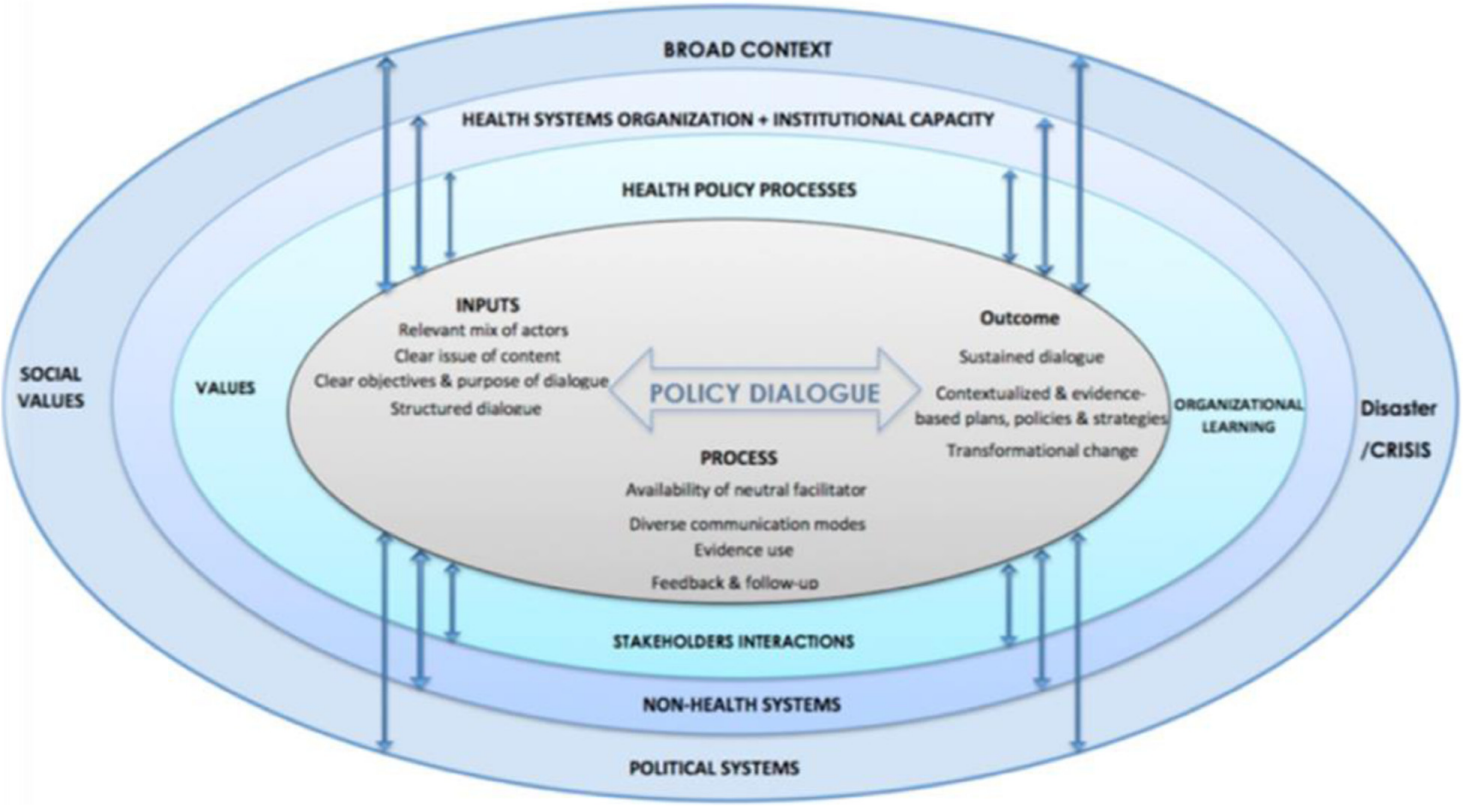
Conceptual framework for health policy dialogue

We conceptualise health policy dialogue as a process concerned with the inputs into the process, the process of dialoguing and the emanating results in the form of policy solutions, referred to as outcomes (see Fig. 1). The process leading to the outcomes is iterative and takes place within the broader health and non-health systems and the broader context of social values and political systems. The process is often characterised by a mix of logistical elements that are conducive to effective dialogues and recognise the values, capacities and power relations that will guide actors' behaviours and interactions [24, 25].

The literature summarises the critical factors for a successful policy dialogue as timely availability of contextualised and relevant evidence; existence of mechanisms for sharing evidence, including views and expertise; secured time and resources to facilitate an open, inclusive and informed discussion among stakeholders; a policy process influenced and shaped by stakeholder inputs; and stakeholders’ monitoring and assessment of the dialogue activities for learning and improvement [1]. The requirements for a successful dialogue have been defined as an evidence-informed, participatory and inclusive interchange aiming for the best policy options; adequate preparation prior to the dialogue, including obtaining consensus on the objectives of the dialogue; a process for participation and sharing of relevant information, identifying actors who will be affected or who will make a significant contribution to the discussion; and appropriate timing of dialogue activities [1].

#### Inputs for a policy dialogue process

When well conducted, a health policy dialogue engages various sectors and diverse stakeholders, giving people a voice in the decision-making process [1]. Scholars emphasise the need to involve actors who (1) can contribute in the generation of a well-informed health policy decision, highlighting that the actors need to have an interest in the policy issues under consideration; (2) have expertise in the political, policy development and group processes; and (3) have the ability to represent relevant stakeholders and their viewpoints [20, 26].^.^ This would be ideal, but the peculiarities of low income countries pose a challenge. Looking at the actors that would be involved, civil society as an example, and it is increasingly playing a significant role in health policy development, but it is faced with difficulties, including the lack of skills, weak internal organisation and inadequate capacity to navigate the political terrain [27, 28]. These limit its ability to meaningfully contribute to the health policy dialogue process. Interaction among the actors is another complicated issue, as it will be influenced by their position, interest in the particular policy issue under discussion and power. Woelk et al. [29] document the challenges in the decision-making process on the use of bednets in three southern Africa countries, where the views advanced by a diverse group of stakeholders were in line with their ideology and commercial interests. This highlights the importance of putting in place mechanisms to identify and manage conflict of interest, if the policy dialogue process is to lead to the most appropriate policy decision [30, 31]. In addition, availability and use of evidence have proven beneficial in reaching technically acceptable decisions on controversial issues [32].

Evidence shows that the objectives and purpose of the dialogue must be clear to guide the discussion towards a given outcome [1, 19], that relevant documentation needs to be shared in advance [20], and that the structure of the dialogue should seek to maximise the contributions of all participants and their interactions [20]. This presupposes that the process will be open and participatory, which may not be the case in some instances. Young [33] points out the limitations peculiar to low income countries, among which are the chaotic nature of policy-making and exaggerated role of donors. There are cases where policy decisions are influenced by donor financing conditionalities despite the existence of policy dialogue structures. Scholars have documented cases where a change in malaria treatment policy was influenced by funding from the Global Fund [34–36], where donor financing conditionalities influenced the change in HIV treatment guidelines [35] and where the use of evidence in policy-making was influenced by donors [36]. Mubyazi et al. [37] document a case in Tanzania where private sector actors resisted a policy change process for malaria treatment because they had invested significantly in the production of choloroquine. Another example is cited by Moat and Abelson [38], where decision-making was driven by high level political offices as opposed to a dialogue through established formal processes. In such instances, the policy dialogue process and subsequent decision-making will be affected by the influence of donors or other influential actors.

Power relations have been shown to affect policy dialogue processes. A case study on the role of civil society organisations in policy dialogue identified three dimensions along which power relations were exercised [39]: (1) visible power emanating from high level power centres such as parliament, legislature and cabinet that guarantee a space for dialogue as a constitutional right; (2) hidden power held by individuals or groups of politicians with vested interests, whose position is crucial for the approval process or funding decisions; and (3) invisible power, which is subtle and includes social, cultural and religious influences. All these affect policy dialogue and policy processes, depending on the issue under consideration. Examples include the case in Uganda where political and cultural issues hampered the development of a policy for medical male circumcision in the efforts to prevent HIV [40]. In that situation, hidden power, manifested through the political stance and the resistance of the communities premised on cultural values, prevented an objective dialogue. Dealing with the different power relations will require different strategies if the dialogue process is to achieve a consensus over a given policy issue and, as such, the power relations have to be anticipated and mitigated.

Another consideration has to do with the roles and responsibilities of the different actors in the health policy dialogue. Ideally the government should take leadership of the policy dialogue process, but there are instances where this has been taken over by other entities. Nabyonga-Orem et al. [41] document a case where a policy dialogue on user fees for health was dominated by the World Bank. Malik et al. [42] also report on an experience from Sudan where a civil society organisation played an instrumental role in changing the malaria treatment policy. This implies that the roles played by the different actors may vary depending on the policy issue under consideration, although this could also result from a gap in the ministry of health leadership. Ensuring government ownership of the national health policies and strategic plans calls for ensured ownership of the health policy dialogue process, and, as such, the relevant capacity must be built.

#### Process of policy dialogue

The elements considered critical for the process of policy dialogue are diverse communication channels, good facilitation, use of evidence, feedback, and follow-up. Use of multiple communication channels is important to ensure that the views of all stakeholders are garnered [19]. Such communication channels include face-to-face meetings, consultations, engagement, advocacy, creation and use of platforms giving preference to country-owned systems [9], and visual aids [20, 43]. Multiple channels will offer better results than any one channel used solely, but this has cost implications that may be difficult to tackle in low income countries. We emphasise, however, that the nature of the problem to be addressed, whether or not people are familiar with the problem, the level of understanding of the actors involved, and the time and resources available to engage in the dialogue should inform the choice of the communication channels.

Neutrality of the facilitator is essential to ensure equal participation and consideration of all actors’ views. A facilitator should be skilful enough to ensure that the discussion is focussed and neutral, in order to ensure open and frank participation [24], as well as have intermediate-level knowledge about the issue under consideration and the local context, in order to manage actors’ contributions and the group dynamics during the deliberations [26]. Lavis et al. [26] consider a neutral facilitator as one who “ensures that participants perceive the dialogue as a ‘safe harbour’ as opposed to a vehicle to steer deliberations in a direction of their preference”. There is need to guard against perceptions of privileged consideration in relation to language, status or resources, among other things. However, in donor-dependent nations, which many of the low income countries are, the exaggerated role of donors, as pointed out by Young [33], will always remain a challenge to the national leadership of the dialogue process. The varied capacity among the actors presents another difficulty.

Challenges in using evidence in decision-making have been documented as well, among which are the limited supply of relevant evidence, the poor quality of the evidence, which is provided in untimely manner, and the weak capacity of the ministry of health to lead the knowledge translation (KT) process [44–47]. Highlighted also are the factors that may be favourable to low income countries, among which are the recognition of KT as a systematic process that starts at the point of setting the research agenda, progresses through the generation of evidence and culminates in the application of the evidence; the ministry of health’s leadership of the KT process; partnerships for KT; and establishment of systematic and institutionalised platforms for engagement [48], a role that policy dialogue platforms may play. Uneke et al. [16] document the use of a health policy advisory committee as a KT platform in Nigeria.

Availability of follow-up and feedback mechanisms is crucial to allow the actors the opportunity to describe the insights they draw from the dialogue or actions they see as critical in addressing a high priority issue, as well as to review the implications of the decisions taken [26]. This may also serve as an avenue for facilitating reporting back of the stakeholders to their constituencies.

#### Outcomes of a health policy dialogue

What the outcomes of a health policy dialogue are and how they can be assessed are other murky areas and will vary depending on the issue under consideration. This notwithstanding, the results of a policy dialogue process, among other things, may include plans, strategies and policy actions that are more comprehensive, consensual and evidence informed than those from traditional processes. Case studies in Bangladesh, Mozambique and Uganda on civil society engagement in policy dialogue identified some planned and unexpected outcomes associated with the different stages of the policy cycle [23]. If indeed policy dialogue is believed to span the whole spectrum from policy-making to monitoring, the outcomes could be assessed in a similar manner. We caution, however, that attribution ought to be made carefully, given the multiplicity of confounding factors that exist. Furthermore, there is need to incorporate a time aspect in the attainment of health policy dialogue outcomes. Although some outcomes may be immediate, for example the generation of evidence that is discussed in a health policy dialogue forum where a decision is taken, other outcomes may be long term, like those affecting behavioural change.

The likelihood of a favourable outcome from a policy dialogue process will be impacted by the characteristics of the issue under consideration [49]. Such characteristics include the extent to which the issue is polarising, that is whether it is likely to cause fragmentation or high polarisation among the actors involved, given their positions on it [49, 50]. In the case of an issue of low polarisation, potential beneficiaries share similar opinions and preferences and they all see the issue as a problem, so the discussions are more likely to be objective and a consensus to be reached. For high polarisation issues, discussions are likely to be entangled in political debates and unbalanced power play. In such instances the outcome of the policy dialogue may be tilted towards the position of the more influential actors [49].

## Conclusion

This article argues that policy dialogue needs to be appreciated as an iterative process that spans the whole process from policy-making to policy implementation, and covering policy review and monitoring and subsequent policy revisions. Institutionalised policy dialogue platforms, an open and participatory policy-making process, ensuring that preparations and logistical arrangements are adequate, mutual respect of stakeholders, and good facilitation are crucial for a good policy dialogue process. There is need for innovative ways of addressing the issues faced by low income countries. The issue under consideration will influence which stakeholders will need to be involved, and, as such, stakeholder mapping needs to be a part of the policy dialogue process. The policy dialogue process needs to be cognisant of the diverse capacities and interests of stakeholders. This calls for capacity building and putting in place mechanisms to manage conflict of interest. Likewise, power relations do impact policy dialogue processes, and they have to be anticipated and mitigated. The different power relations will require different strategies if the policy dialogue process is to achieve a consensus over a given policy issue. The likelihood of a favourable outcome from a policy dialogue process will be impacted by the characteristics of the issue under consideration and whether it is contested or not, and the policy dialogue process needs to be tailored accordingly.

## Abbreviations

GHI, Global health initiative; KT, knowledge translation; MDG, millennium development goal; WHO, World Health Organization
